# Decrypting the potency of anti-cancer therapeutics by using mass spectrometry to quantify post-translational modifications

**DOI:** 10.1016/j.crmeth.2023.100483

**Published:** 2023-05-22

**Authors:** Sophie A. Herbst, Forest M. White

**Affiliations:** 1 Department of Biological Engineering, Massachusetts Institute of Technology, Cambridge, MA 02139 USA; 2 Center for Precision Cancer Medicine, Koch Institute for Integrative Cancer Research, Massachusetts Institute of Technology, Cambridge, MA 02139, USA

## Abstract

In a recent issue of *Science*, Zecha et al.^1^ present decryptM, an approach aimed at defining the mechanisms of action of anti-cancer therapeutics through systems-level analysis of protein post-translational modifications (PTMs). By using a broad range of concentrations, decryptM generates drug response curves for each detected PTM, enabling identification of drug effects at different therapeutic doses.

## Main text

Post-translational modifications (PTMs) are essential for regulating protein function and activity in biological systems. PTMs can influence protein activity, interactions, stability, and subcellular localization, making them critical for cellular processes such as signaling, gene expression, and metabolism. Moreover, PTMs are known to play a crucial role in the response of cells to therapeutic compounds.^2^

Despite this, only few studies investigating the effects of therapies on PTMs exist and research has often been limited to proteins with available antibodies against PTMs. Many therapeutic compounds exert polypharmacology, e.g., multiple proteins are directly affected by the therapy. Inability to comprehensively analyze PTMs thus limits our understanding of the complex interplay between therapies and cellular pathways.

These limitations can be overcome by using mass spectrometry to analyze PTMs. For this analysis, samples are left untreated or are treated with a particular compound. Following lysis, protein digestion, purification, and optional multiplexing, samples are subjected to a protocol enriching for peptides containing the modification of interest., e.g., protein phosphorylation, ubiquitination, or acetylation. The enriched peptides are then analyzed by mass spectrometry. Mass spectrometry-based profiling of PTMs allows for the simultaneous detection and quantification of thousands of PTMs. Previous studies have employed mass spectrometry to analyze PTMs regulated by therapeutics and showed that this approach can give insights into the response of cells to treatment, and that these could potentially have clinical implications, e.g., van Beijnum, et al.^3^ Lopez, et al.,^2^ Kohale et al.,^4^ Porras-Yakushi et al.,^5^ and Solanki et al.^,^^6^

However, due to the time and costs involved when generating such datasets, most existing studies limited their work to a single dose per compound.

**Figure 1:**
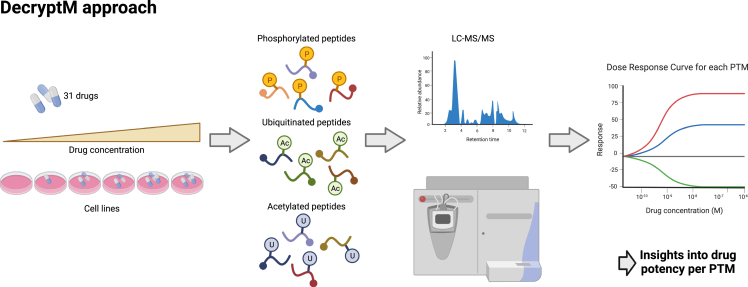
Schematic representation of the DecryptM workflow The decryptM approach described by Zecha et al.^1^ uses mass spectrometry-based profiling of post-translational modifications (PTMs) to study drug mechanisms of action. Cell lines are treated with multiple concentrations of a therapeutic and processed for mass spectrometry. Enrichment of the PTMs of interest is performed prior to analysis with liquid chromatography-tandem mass spectrometry (LC-MS/MS). The concentration-resolved data allows for the generation of a drug response curve for each detected PTM, thus generating hypotheses regarding the potency of a compound to regulate specific PTMs. Figure created using BioRender (https://biorender.com).

In a study published in *Science*, Zecha et al.^1^ have extended this approach by profiling anti-cancer drugs at multiple different concentrations. Using their workflow, which they call decryptM (Figure 1), the authors analyzed the phosphoproteome, ubiquitinome, or acetylome to generate drug response curves and EC50 values for each detected PTM for a set of 31 anti-cancer compounds. Based on the EC50 values, the authors hypothesized how potently each compound affects the particular PTM. They also demonstrated the potential to create time-resolved data and tested their approach in a total of 13 cell lines.

To validate that low EC50 values correspond to a higher potency of therapeutics toward a certain PTM, Zecha et al.^1^ examined direct binding partners of the tested compounds by using kinobeads, Sepharose beads with broadly selective kinase inhibitors bound to the surface. Incubation of kinobeads with cell lysate in the presence or absence of a specific inhibitor of interest, and subsequent analysis by mass spectrometry, can determine the affinity of proteins toward the specific inhibitor versus the bound unspecific kinase inhibitors on the beads.^7^ Kinobead analysis allows the identification of direct binding partners of a certain compound in the context of a complex environment. By comparing the EC50 values obtained by decryptM with the binding potency of proteins to compounds obtained with kinobeads, the authors were able to confirm that the PTM-specific responses to the therapeutics reflected the drug-target affinities. These findings demonstrated that the generated drug response curves and EC50 values for PTMs can provide valuable information about the mechanisms of drug action.

Proof-of-concept applications of decryptM include highlighting the specific effects of several therapeutics on different cell lines, including chemotherapeutic drugs, protein interaction inhibitors, proteasome inhibitors, epigenetic drugs, kinase inhibitors, and antibodies. For chemotherapeutic drugs, highly specific alterations could be detected upon treatment within 30 min, including expected responses such as altered DNA damage response as a result of cytarabine treatment. Using proteasome inhibitors as an example, the authors demonstrated the applicability of their approach to generate time-resolved data. Prolonged treatment with the inhibitors bortezomib or carfilzomib increased the cell’s stress response, as determined by phosphoproteomics, and resulted in elevated levels of protein ubiquitination. Moreover, Zecha et al.^1^ showed that acetylation profiles, upon treatment with lysine deacetylases, closely mirrored the target specificity of the individual compounds. Additionally, the authors used decryptM to assess PTM targets of 10 kinase inhibitors and compared the induced phosphorylation patterns, highlighting similarities and differences between the compounds. Similar to the study published by Schwill et al.,^8^ but using more drug concentrations and three HER2 or HER3-positive breast cancer cell lines, Zecha et al.^1^ employed their mass spectrometry-based workflow to study the effect of compounds blocking HER2 on PTMs. Even though the kinase inhibitor lapatinib and the antibodies trastuzumab and pertuzumab all have the same main target, Zecha et al.^1^ showed that each individual agent had a unique set of affected phosphorylation sites. In accordance with the literature, the authors did not see any blocking of mitogenic signaling by trastuzumab, which is in contrast to the effects observed in lapatinib-treated cells. Finally, they used their approach to study how rituximab, a monoclonal antibody used to treat certain types of B cell malignancies, kills cells. They found that treatment triggers a dominant signature for activation of B cell receptor signaling, as well as cellular apoptosis in rituximab-sensitive cells, in agreement with previously published results.^9^

Through these proof-of-concept studies, the authors have generated a massive PTM data compendium that they could only briefly discuss in the limited length of their publication. The data uncovered many PTMs that were altered upon drug treatment. In their manuscript, Zecha et al.^1^ focused on describing PTMs which could help them to decipher the mechanism of action of a certain compound. Analyses looking at PTMs which are regulated opposite to the expected direction could complement these findings and provide insights into adaptive responses by the cells trying to circumvent cell death. Moreover, motif analysis of co-regulated PTMs might allow for the identification of enzymes that regulate them. Additional correlation and clustering of PTMs to build a substrate-enzyme network could provide insights into substrate-enzyme relationships and help in the discovery of new putative substrates.

In combination with methods probing drug-target affinities, such as kinobeads^7^ or thermal proteome profiling (TPP),^10^ decryptM data may enable further hypothesis generation regarding direct and indirect targets of compounds, while also providing information on downstream signaling networks associated with drug-target interactions. Such information could aid in the development of more targeted treatments or of combination therapies attempting to avoid the emergence of resistance.

Zecha et al.^1^ do not validate the claims in the paper by follow-up experiments and, contrary to their statement emphasizing the importance of the cell line context for drug response, they profile only one cell line per therapeutic for 80% of the analyzed compounds. Despite these shortcomings, the decryptM approach and the resulting dataset is a powerful resource for biologists investigating drug actions. It has potential to serve as a valuable hypothesis-generating tool, especially as all data are publicly available on PRIDE (PXD037285) and integrated into ProteomicsDB. Extending the dataset with further compounds, cell lines, and time points, and improving its accessibility by, for example, creating an interactive web application to make the exploration of drug response curve more easily accessible, would improve the impact of their research.

In conclusion, decryptM offers a promising approach for investigating the effects of therapeutics on PTMs, with potential implications for drug discovery and personalized medicine. Although further validation of the predictions is necessary, the dataset provides a valuable tool for hypothesis generation and the data generated by this study holds a wealth of potential for further investigation.
